# Self-Assembled Nanostructures in Aprotic Ionic Liquids Facilitate Charge Transport at Elevated Pressure

**DOI:** 10.1021/acsami.3c08606

**Published:** 2023-08-09

**Authors:** Beibei Yao, Marian Paluch, Jaroslaw Paturej, Shannon McLaughlin, Anne McGrogan, Malgorzata Swadzba-Kwasny, Jie Shen, Beatrice Ruta, Martin Rosenthal, Jiliang Liu, Danuta Kruk, Zaneta Wojnarowska

**Affiliations:** † Faculty of Science and Technology, Institute of Physics, University of Silesia in Katowice, 75 Pułku Piechoty 1A, 41-500 Chorzów, Poland; ‡ The QUILL Research Centre, School of Chemistry and Chemical Engineering, The Queen’s University of Belfast, David Keir Building, Stranmillis Road, BT9 5AG Belfast, NI, U.K.; § Institut Neel, 38000 Grenoble, France; ∥ ESRF—The European Synchrotron, CS 40220, 38043 Grenoble, France; ⊥ Department of Chemistry, KU Leuven, Celestijnenlaan 200F, Box 2404, B-3001 Leuven, Belgium; # Dual Belgian Beamline (DUBBLE), European Synchrotron Radiation Facility, 71 Avenue des Martyrs, CS 40220, 38043 Grenoble Cedex 9, France; ¶ Faculty of Mathematics and Computer Science, University of Warmia and Mazury in Olsztyn, Sloneczna 54, Olsztyn PL-10710, Poland

**Keywords:** ionic liquids, self-assembly, liquid−liquid phase transition, high pressure, charge transport mechanism

## Abstract

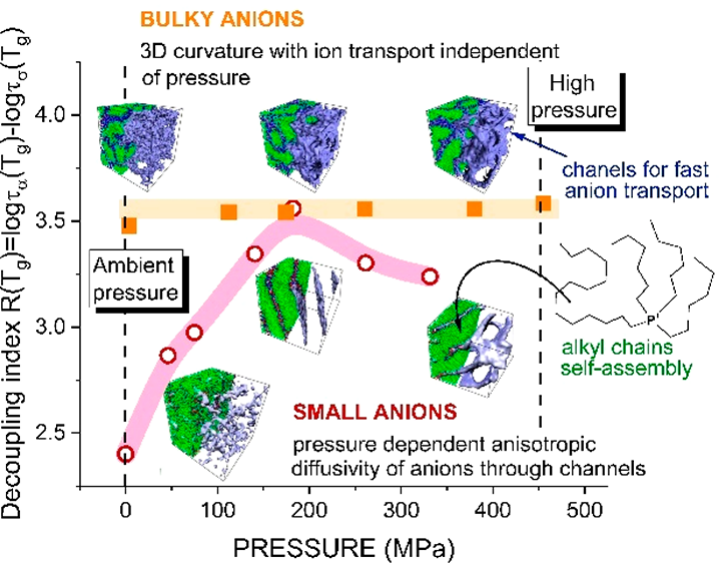

Ionic liquids (ILs), revealing a tendency to form self-assembled nanostructures, have emerged as promising materials in various applications, especially in energy storage and conversion. Despite multiple reports discussing the effect of structural factors and external thermodynamic variables on ion organization in a liquid state, little is known about the charge-transport mechanism through the self-assembled nanostructures and how it changes at elevated pressure. To address these issues, we chose three amphiphilic ionic liquids containing the same tetra(alkyl)phosphonium cation and anions differing in size and shape, i.e., thiocyanate [SCN]^−^, dicyanamide [DCA]^−^, and tricyanomethanide [TCM]^−^. From ambient pressure dielectric and mechanical experiments, we found that charge transport of all three examined ILs is viscosity-controlled at high temperatures. On the other hand, ion diffusion is much faster than structural dynamics in a nanostructured supercooled liquid (at *T* < 210 ± 3 K), which constitutes the first example of conductivity independent from viscosity in neat aprotic ILs. High-pressure measurements and MD simulations reveal that the created nanostructures depend on the anion size and can be modified by compression. For small anions, increasing pressure shapes immobile alkyl chains into lamellar-type phases, leading to increased anisotropic diffusivity of anions through channels. Bulky anions drive the formation of interconnected phases with continuous 3D curvature, which render ion transport independent of pressure. This work offers insight into the design of high-density electrolytes with percolating conductive phases providing efficient ion flow.

## Introduction

Ionic liquids (ILs) are a novel class of fluids showing rich structural diversity in the nature of ions, their interactions, and the organization of ionic species in the liquid phase.^1,2^ Almost unlimited combinations of cations and anions enable tailoring ILs for numerous applications across multiple disciplines in science and engineering, e.g., pharmacy,^3^ chemical synthesis,^4^ and electrochemistry.^5,6^ However, in most instances, the physical arrangement of ions in bulk strongly affects the mechanical and conducting properties of IL, thereby determining process efficiency.^7^ There is growing evidence that the ability to support self-assembly is widespread among ILs, providing another common trait of ionic fluids.^8^ Therefore, a deep understanding of ILs self-assembly is essential to control their properties and, thus, functions comprehensively.

The nanostructure of IL is driven by the spontaneous solvophobic segregation of charged and uncharged groups into polar and apolar domains.^7^ Thus, amphiphilic ions with both polar and apolar moieties, like localized and exposed charges or long alkyl chains, have a stronger tendency to self-assemble. If the ions are weakly amphiphilic, the bulk structure is determined mainly by Coulombic forces and simple packing.^9^ Hence, the degree of nanostructure in ILs usually scales with the elongation of a cation alkyl chain, while anions control local structure in the polar domains. For instance, imidazolium and pyrrolidinium-based ILs develop amphiphilic nanostructure (like micelles) above *n*-butyl chains.^10,11^ However, the geometry of trialkylmethylammonium (N_1,nnn_^+^) cation is sufficient to facilitate the nanostructure formation even with short ethyl tails.^12^ These studies emphasize the importance of the volume ratio of charged and uncharged groups (*V*_alkyl_:*V*_polar_) and the relative positions of the alkyl groups on the cation. In principle, for a larger *V*_alkyl_:*V*_polar_ ratio, stronger segregation of polar and apolar domains is observed.^13^ Ions forming hydrogen-bonded networks also support amphiphilic self-assembly.^14^ Thereby, nanostructures are observed in both protic and aprotic ILs.^15^

Over the years, numerous experimental techniques and theoretical studies have been focused on the effect of temperature on the nanoscale morphology of ILs.^16−18^ It has been reported that thermal fluctuations make ILs homogeneous fluids without any self-assembly behavior at high temperatures. On the other hand, cooling brings competition between hydrophobic and hydrophilic interactions accompanied by changes in ions conformation. Various nanoscale phases arise as the molecules rearrange in a supercooled liquid state.^11^ In this context, it should be noted that isobaric cooling decreases the kinetic energy and increases the ions’ packing (density), making the self-assembly complex and puzzling. Therefore, the combined effect of thermal and density fluctuations must be separated to thoroughly understand IL (nano)structure formation. It can only be achieved by performing high-pressure experiments.^19^ Since simple packing constraints determine the general arrangement of polar and nonpolar domains, the effect of pressure on ions rearrangement is naturally expected. Since the pressure-dependent experiments are far more complex than their temperature counterpart, there are no reports on the behavior of ILs self-assembled nanostructures under pressure. A methodology that can address this challenge employs high-pressure dielectric spectroscopy.^20^ In addition to insight into dc-conductivity behavior and relaxation dynamics at various *T*–*P* conditions, isothermal dielectric measurements offer a unique possibility to recognize the dominating charge transport mechanisms in low-molecular ILs and their polymer counterparts.^21−24^ Thereby, high-pressure dielectric experiments can capture two missing crucial aspects: ion dynamics through the self-assembled nanostructures and how it changes at elevated pressure.

The current paper discusses the conductivity mechanism as a function of the structural organization of three trihexyl(tetradecyl)phosphonium [P_666,14_]^+^-based aprotic ionic liquids over a wide temperature and pressure range. Specifically, the [P_666,14_]^+^ cation has been combined with three structurally different anions: thiocyanate [SCN]^−^, dicyanamide [DCA]^−^, and tricyanomethanide [TCM]^−^. According to X-ray diffraction and FT-IR spectroscopic studies of borohydride IL [P_666,14_][BH_4_], presented in ref (25), enhanced ordering of alkyl chains occurs in the supercooled liquid state accessible through the first-order liquid–liquid phase transition (LLT). Our ambient pressure dielectric and mechanical measurements covering both supercooled liquids and glassy state reveal high similarities in the relaxation dynamics of all three studied systems. Specifically, at high temperatures (*T* > *T*_LL_), the charge transport is fully controlled by viscosity (so-called vehicle mechanism), while in a supercooled state revealing self-assembly behavior (*T* < *T*_LL_), ion diffusion is much faster than the structural dynamics. Furthermore, the conducting properties of self-assembled glass differ from typical IL and depend on the thermal history of sample. To disclose the details of the charge transport mechanism in [P_666,14_]^+^ materials, high-pressure dielectric spectroscopy has been employed. High-pressure experiments combined with MD simulations show that the nanostructure of [P_666,14_]^+^-ILs depends on the anion size and reveals different pressure sensitivities for each material.

## Materials and Methods

The synthesis of examined [P_666,14_][SCN] and [P_666,14_][TCM] is provided in Supporting Information. [P_666,14_][DCA] with a purity of 99% was supplied by Iolitec.

### Differential Scanning Calorimetry (DSC)

Calorimetric experiments of studied ILs were performed by means of a Mettler Toledo DSC1STAR system equipped with a liquid nitrogen cooling accessory and an HSS8 ceramic sensor (a heat flux sensor with 120 thermocouples). During the experiments, the flow of nitrogen was kept at 60 mL min^–1^. Enthalpy and temperature calibrations were performed using indium and zinc standards. Low-temperature verification was made using *n*-heptane (182.15 K, 140.5 J g^–1^) at different scanning rates (0.7, 1, 5, and 10 K min^–1^). The baseline was constructed as a straight line from the onset to the end point. A dedicated software Mettler Toledo DSC1STAR allows various calculations (onset, heat, peak temperature, etc.) from the original recorded DSC curves. Before the measurement, the samples were annealed for 30 min at 373 K. Temperature ramps involved cooling to 143 K and heating to 373 K with a rate of 10 K/min. Samples were cycled at least 3 times to ensure reproducibility and high accuracy. The 6 h aging experiment was performed at 187 K after cooling with the rate of 10 K·min^–1^.

### Dielectric Measurements

The dielectric measurements at ambient pressure for studied ILs were carried out over a frequency range from 10^–1^ to 10^7^ Hz by means of a Novo-Control GMBH Alpha dielectric spectrometer. The Novocool system controlled the temperature with an accuracy of 0.1 K. During this measurement, the sample was placed between two stainless steel electrodes (diameter = 15 mm). The quartz ring provided the distance between plates. We used the capacitor filled with the studied sample for the pressure-dependent dielectric measurements, which was next placed in the high-pressure chamber and compressed using silicone oil. Note that the sample was only in contact with stainless steel during the measurement. The Unipress setup measured the pressure with a resolution of 1 MPa. The temperature was controlled within 0.1 K by means of a Weiss fridge. To avoid cold crystallization and maintain the same history for each sample, each [P_666,14_]-based IL was quenched to 201 K and then compressed to the glassy state. Afterward, the temperature was increased, and the dielectric data were collected on isothermal decompression. This procedure enables studies of ion dynamics in the glassy and supercooled liquid 2 state. Further decompression to liquid 1 resulted in cold crystallization of [P_666,14_][SCN] and [P_666,14_][DCA] and required melting of the sample in the subsequent step.

### Viscosity Measurements

The viscosity was measured by employing an ARES G2 rheometer. In the supercooled liquid region, aluminum parallel plates of diameter 4 mm were used. The rheological experiments were performed in the frequency range from 0.1 to 100 rad·s^–1^ (10 points per decade) with strain equal to 0.1% in the vicinity of the liquid glass transition. The strain was increased by 1 order of magnitude with every 10 K. The relative uncertainty of the reported viscosity measurements from calibration, temperature, and pressure control, as well as sample purities, did not exceed 7%.

### Small-Angle and Wide-Angle X-ray Scattering (SAXS, WAXS)

To investigate the microscopic structural changes during LLT on a larger scale, we employed X-ray diffraction (XRD) characterization with an energy of 12 keV. Small-angle X-ray scattering (SAXS) measurements were performed in the *q* range of 0.13–0.8 Å, while wide-angle X-ray scattering (WAXS) measurements were conducted in the *q* range of 0.6–4.9 Å. The measurements were performed on cooling and subsequent heating, and the two-dimensional data were acquired by using independent detectors. The one-dimensional data were obtained by azimuthal integration using the pyFAI program. The temperature of the system was controlled by Linkam, and the cooling/heating rate was set to 10 K/min.

### NMR Relaxometry Studies

The ^1^H spin–lattice relaxation data of [P_666,14_][DCA] were collected by a STELAR fast field cycling (FFC) relaxometer in the frequency range of 10 kHz to 25 MHz at 204 and 201 K. Data analysis has been performed with the following parameters: ρ = 0.90 7 g/cm^3^, *M* = 549.90 g/mol, *N*_H_ = 6.75 × 10^28^/m^3^.

### MD Simulations

Molecular dynamics simulations of ionic liquids comprised of amphiphile molecules were performed using a bead–spring model at coarse-grained resolution. Details on the adopted numerical model are included in Supporting Information.

## Results and Discussion

### Samples Characterization

**Figure 1:**
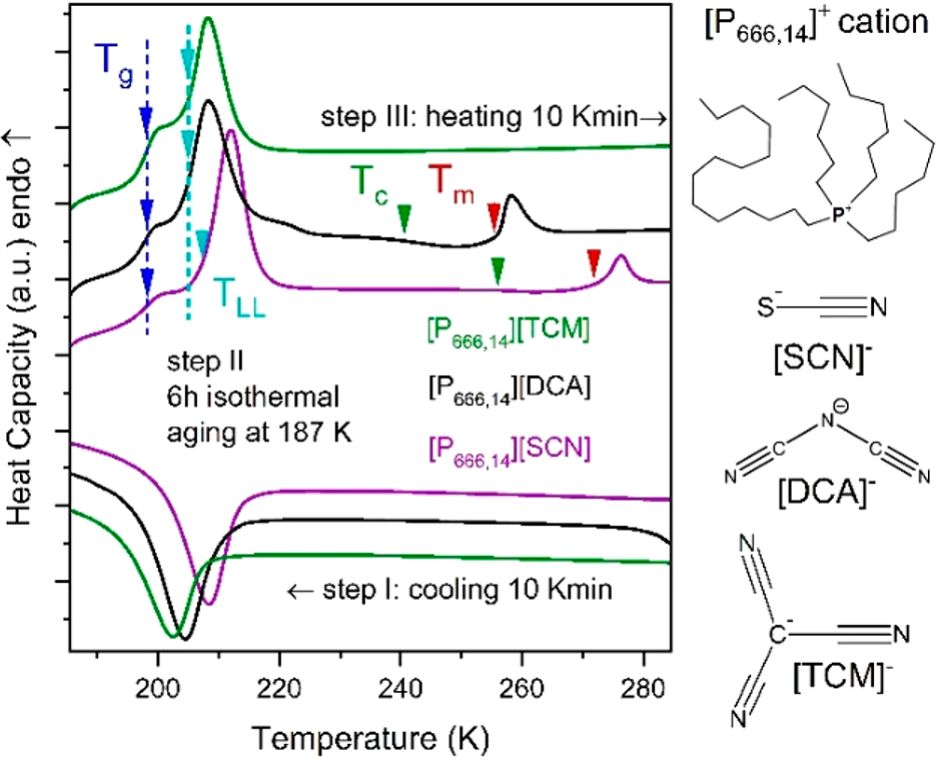
Differential scanning calorimetry (DSC) traces of [P_666,14_]^+^-based ILs. Arrows indicate the onset of LLT (cyan), melting point (red), cold crystallization (green), and *T*_g_ (blue). The values of liquid–liquid transition temperature (*T*_LL_), onset of cold crystallization (*T*_c_), melting temperature (*T*_m_), and enthalpy of these processes Δ*H* are collected in Table S1. The chemical structures of the [P_666,14_]^+^ cation and [TCM]^−^, [DCA]^−^, and [SCN]^−^ anions are also presented.

The materials examined herein were chosen as model systems to understand the effect of molecular architecture and external thermodynamic variables (temperature and pressure) on the charge transport mechanism in amphiphilic aprotic ionic liquids, revealing self-assembly behavior. Specifically, the [P_666,14_]^+^ cation with long alkyl chains has been combined with three anions of different sizes and shapes: a relatively small linear thiocyanate, [SCN]^−^, a larger dicyanamide with bent geometry, [DCA]^−^, and the largest, trigonal planar tricyanomethanide, [TCM]^−^. Due to the amphiphilic nature of the [P_666,14_]^+^ cation, the chosen ILs are expected to reveal self-assembly behavior. Recent X-ray diffraction and FT-IR spectroscopic studies of the borohydride salt [P_666,14_][BH_4_] confirmed the enhanced ordering of long alkyl chains in a supercooled liquid state accessible through the first-order liquid–liquid phase transition. Consequently, two liquid phases have been found within single component material: first, at *T* > *T*_LL_ (liquid 1) and the second with nanoscale structuration at *T* < T_LL_ (liquid 2). The LLT has also been reported for [P_666,14_][SCN] and [P_666,14_][TCM];^26^ however, the physical properties of [P_666,14_][DCA] were studied only at room temperature conditions (thermal stability, viscosity, and dc-conductivity).^27^ To bridge this gap, first, differential scanning calorimetry (DSC) studies of [P_666,14_][DCA] have been carried out, and collected thermograms were compared to data recorded for [P_666,14_][SCN] and [P_666,14_][TCM] in Figure 1. As presented, cooling at the rate of 10 K·min^–1^ results in a broad exotherm with onsets at 207.4, 209, and 213.3 K for [P_666,14_][TCM], [P_666,14_][DCA], and [P_666,14_][SCN]. Subsequent heating performed after the time-dependent isothermal step at 187 K (the so-called aging process) revealed the step-like change of heat capacity, followed by a well-resolved endothermic peak, the first indicating liquid-glass transition (*T*_g_ = 197 K the same for all examined here ILs) and the latter, reversible with respect to cooling scan, denoting the first-order liquid–liquid transition (LLT). More thermal effects were observed upon further heating of [P_666,14_][DCA] and [P_666,14_][SCN]: the onset of cold crystallization (*T*_c_) and subsequent melting (*T*_m_). However, due to the small enthalpy of these events, one can conclude that only partial crystallization of these two ILs occurred upon heating at a standard rate of 10 K·min^–1^. Extending the time for nucleation and crystal growth by decreasing the heating rate makes both *T*_c_ and *T*_m_ more detectable, while the onset of LLT remains the same (see Figure S1 and Table S1). The same experimental protocol applied to [P_666,14_][TCM] shows a strong resistance to crystallization.

**Figure 2:**
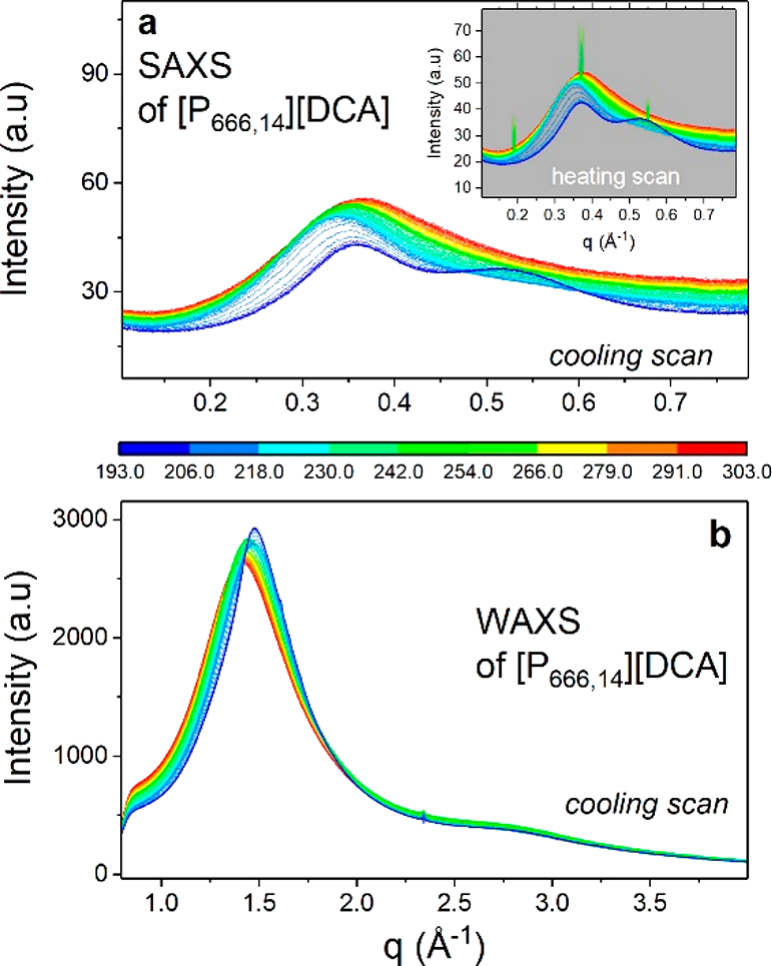
X-ray scattering intensity of [P_666,14_][DCA] at various temperatures recorded on cooling and subsequent heating (inset). Panel a presents SAXS results of [P666,14][DCA], while panel b presents WAXS data of the same IL.

To investigate the structural changes accompanying LLT, XRD measurements were performed. Figure 2 shows the temperature-dependent structure functions obtained from experiments for [P_666,14_][DCA]. As can be seen, the examined IL shows three characteristic peaks at values of *q* below 2 Å^–1^. According to literature reports,^28^ the most intense diffraction peak near 1.4 Å^–1^ arises from a short-range separation between counterions combined with a carbon–carbon interaction from cation alkyl chains. The latter indicates that hydrophobic tails of [P_666,14_]^+^ have significant contact. This intermolecular peak shifts toward higher *q* values with cooling due to the increase in density and becomes noticeably narrow when IL enters the liquid 2 state. At the same time, the intensity of the first diffraction peak, the so-called prepeak (0.36 Å^–1^ at RT) identified with long-range anion–anion correlations, becomes smaller with decreasing temperature and the other peak starts to appear on its high-*q* side when the liquid 2 state is achieved. The latter one, observed finally at around 0.53 Å, can be due to the separation of ions of the same charge. The subsequent heating brings opposite changes in the XRD pattern. However, when the temperature rises above *T*_LL_, the Bragg peaks indicating cold crystallization of [P_666,14_][DCA] appear (see inset of Figure 2a). These results correspond well with the DSC thermograms discussed above.

### Charge Transport Mechanism above and below the *T*_LL_

**Figure 3:**
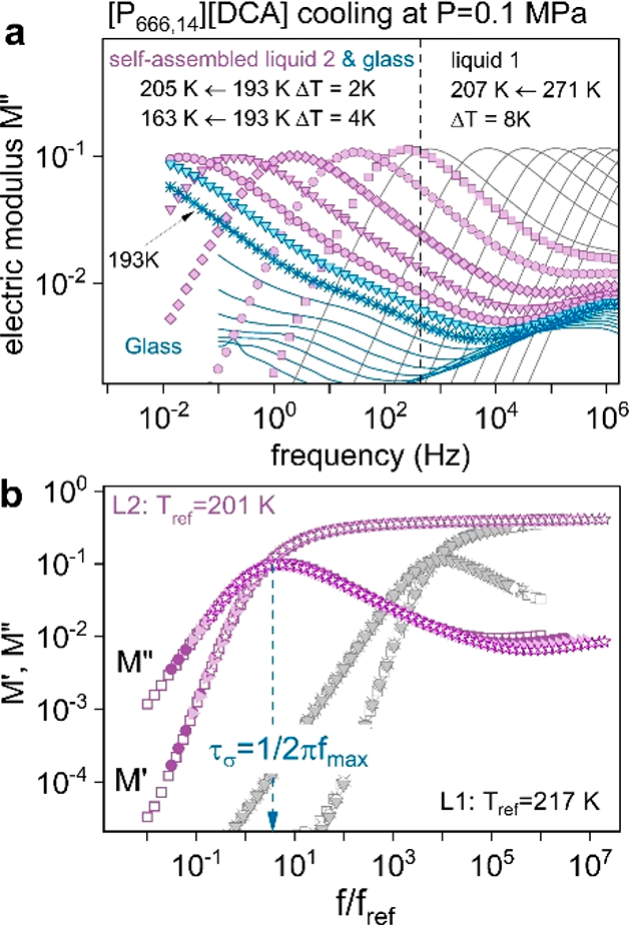
Dielectric response of [P_666,14_][DCA] under ambient pressure conditions. (a) Representative dielectric data of [P_666,14_][DCA] in glass (blue scatters and lines), liquid 2 (violet scatters), and liquid 1 (solid lines) phases were obtained on cooling. (b) Representative dielectric data of [P_666,14_][DCA] measured in the supercooled liquid 1 state (gray symbols) and self-assembled liquid 2 state (violet symbols) superimposed to each other.

In the next step, dielectric measurements were performed to examine the charge transport mechanism across the LLT and near the liquid–glass transition. Two experimental protocols have been applied to realize this task. In the first one, the dielectric data were collected on cooling, which allowed for monitoring changes in ion dynamics during the transition from a simple liquid state to a nanostructured one. In the second procedure, the ionic liquids were quenched to 187 K (corresponding to a glassy state) in the dielectric setup, and then frequency scans (10^–2^–10^6^ Hz) were recorded upon heating at Δ*T* = 1 K intervals, i.e., with the rate of 1 K min^–1^. A complex electric modulus, M*(*f*) = ε*(*f*)^−1^ = *M*′(*f*) + i*M*″(*f*), was adopted to analyze the dielectric response of examined systems. Representative results for [P_666,14_][DCA], in the form of the imaginary part of the M*(*f*) function, over a broad range of temperatures, are depicted in Figure 3a. The modulus peak position, *f*_max_, is strongly temperature-dependent and shifts toward lower frequencies with cooling, which is a typical behavior of ILs. This indicates slower ion mobility and a longer time scale of charge transport in a given system at lower temperatures. At a specific temperature close to *T*_LL_^DSC^, the amplitude of the *M*″(*f*) function decreases slightly and then maintains a new level.

At the same time, the *M*″(*f*) peak becomes broader (see also Figure 3b). Below 195 K, the *M*″(*f*) peak, frequently called σ-relaxation, moves out of the experimental window. Then, a secondary mode characterizing the dynamics of the glassy state appears. Analogous results have been observed on the heating scan (Figure S2); however, an increase in temperature above *T*_LL_^DSC^ caused cold crystallization. Using the same experimental protocol, that is, heating of quenched IL, we observed cold crystallization of [P_666,14_][SCN] in phase 1, whereas phase 2 was stable. At the same time, both liquid states of [P_666,14_][TCM] were thermodynamically stable (Figure S3). These results stay in agreement with previously described DSC data.

**Figure 4:**
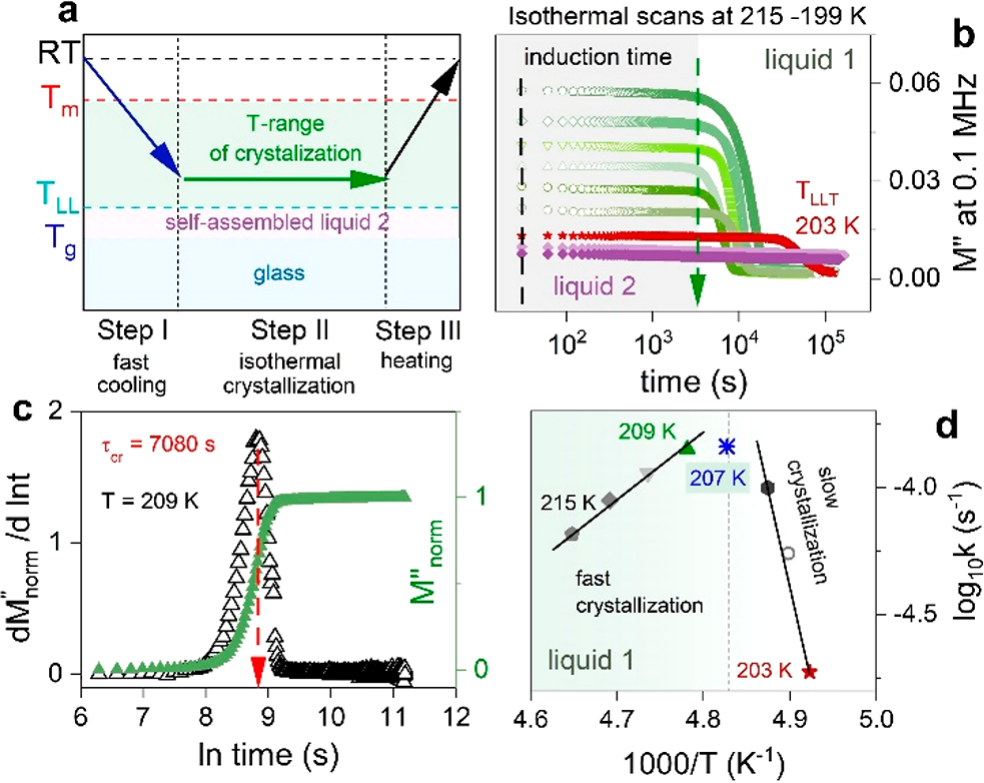
(a) The isothermal-time-dependent dielectric measurements were performed in the crystallization range. Each scan was started after a quench from RT. (b) Time evolution of M″ at 0.1 MHz and various temperatures in liquid 1 and liquid 2 states of [P_666,14_][DCA]. (c) Representative kinetic curve of [P_666,14_][DCA] obtained from data presented in panel b and normalized using the *M*_norm_^″^(*f*) =  (right axis). Avrami–Avramov plot constructed for [P_666,14_][DCA] at 209 K (left axis). (d) Rate constant *k* of the crystallization process as a function of inverse temperature.

To characterize the physical stability of supercooled liquid 1 and liquid 2 states of [P_666,14_][DCA] thoroughly, the time-dependent dielectric scans within the *T* range 199–215 K were taken after quench-cooling from room temperature. The procedure for isothermal dielectric measurements is schematically presented in Figure 4a. Subsequently, single-frequency (0.1 MHz) time-dependent scans of [P_666,14_][DCA] were performed. As shown in Figure 4b, at least 1.5 h of induction time is required to start a cold crystallization process at any studied *T* in the liquid 1 state. Later on, a decrease of *M*″ over time takes place. Contrary, phase 2 of [P_666,14_][DCA] is a stable liquid without crystallization tendency (see violet lines in Figure 4b). To characterize the crystallization of phase 1 quantitatively, the normalized electric modulus *M*_norm_″(*f*) has been analyzed in terms of the Avrami–Avramov model. The representative Avram–-Avramov plot for crystallization kinetics at *T* = 209 K is shown in Figure 4c.

According to the Avramov model, the maximum value of d*M*″_norm_/d(ln *t*) vs ln *t* gives the characteristic time of crystallization τ_cr_ that is inversely related to crystallization rate *k* = 1/τ_cr_. The latter, plotted in log scale vs *T*^–1^, indicates two distinct regions of differing propensity to crystallize in liquid 1 state; first with the activation energy of ∼100 kJmol^–1^ close to the LLT and second with *E*_a_ = 45 kJ/mol far from the LLT (see Figure 4d).

To describe the dynamics in both supercooled liquids and the glassy state, we chose a frequency point where the *M*′(*f*) and *M*″(*f*) crossed each other (*f*_cross_) and determined the time scale of conductivity (σ) relaxation (τ_σ_ = 1/(2π*f*_cross_)) over a wide temperature range, covering both liquid 1 and liquid 2. Note that *f*_cross_ corresponds perfectly to *f*_max_. To extract the value of conductivity relaxation, τ_σ_, in the *T*_g_ region, σ-peak recorded at 197 K has been shifted horizontally to the temperatures *T* < *T*_g_ so that its high-frequency side superimposes with the spectra collected in the glassy state. This operation could be employed since all conductivity relaxation modes in liquid 2 retain the same shape; i.e., the time–temperature superposition (TTS) rule is satisfied (Figure 3b).

**Figure 5:**
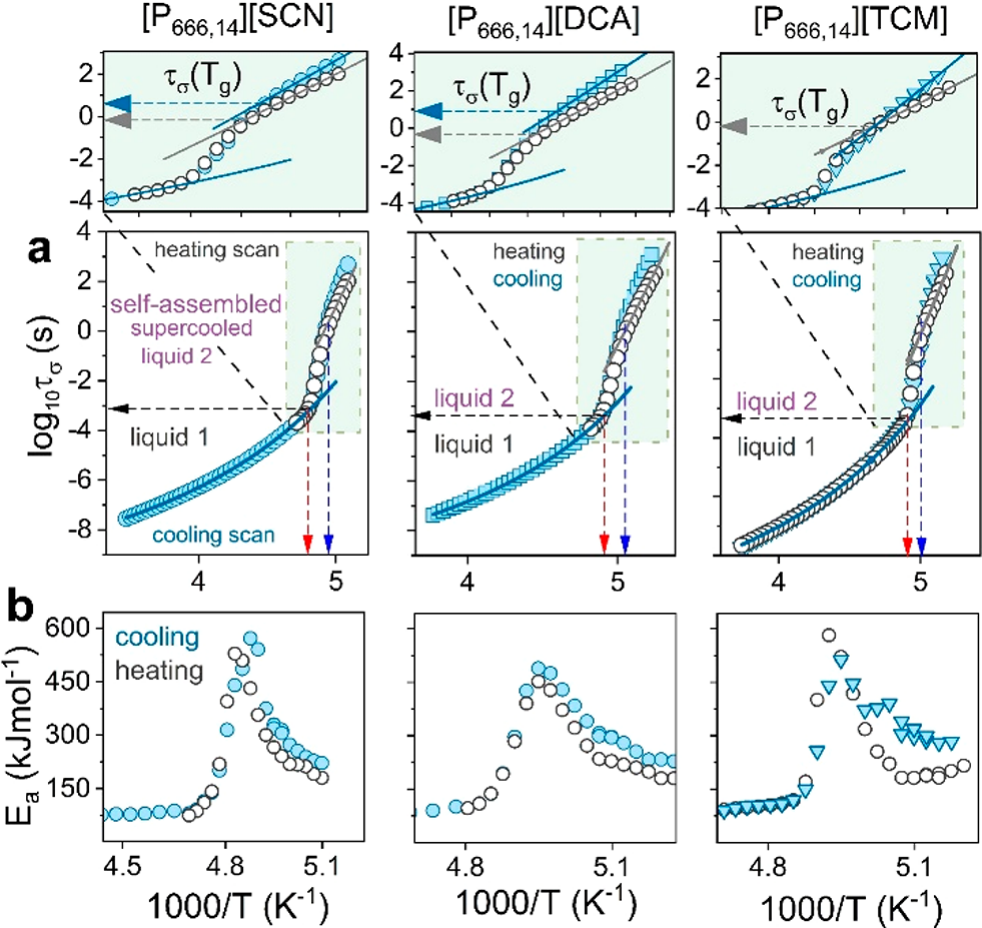
(a) Comparison between the temperature dependence of conductivity relaxation time for [P_666,14_][SCN], [P_666,14_][DCA], and [P_666,14_][TCM] (from left to right) obtained cooling and heating scans. Heating has been performed after the quench cooling to the glassy state. Scatters indicate experimental data, and solid lines in liquid 1 state denote the fit of VFT function τ_σ_ =  to experimental data. Dashed lines indicate *T*_g_ and the temperature of LLT (blue and red arrows). Zooms highlight different τ_σ_(*T*_g_) obtained on cooling (blue arrow) and heating (gray arrow) scans. (b) Apparent activation energy *E*_a_ =  calculated from dielectric data obtained on cooling and heating for [P_666,14_][SCN], [P_666,14_][DCA], and [P_666,14_][TCM].

As illustrated in Figure 5a, all three physical states are well identified on the relaxation map of [P_666,14_][DCA], [P_666,14_][SCN], and [P_666,14_][TCM]. Liquid 1 reveals a VFT-type behavior that changes toward Arrhenius law, τ_σ_ ∝ exp[−*E*_a_/(*k*_B_*T*)], at *T*_LL_^DSC^. A substantial increase in activation energy accompanies this behavior (Figure 5b).

Upon vitrification of supercooled liquid 2 taking place at *T*_g_^DSC^, the τ_σ_(*T*^–1^) continues the Arrhenius dependence but with much lower activation energy (see the zoom). The crossover of τ_σ_(*T*^–1^) visible at *T*_g_ is an inherent part of the liquid–glass transition and reflects the slowing down of charge transport in a disordered solid state.^29^ However, close inspection of Figure 5a reveals that the time scale of charge diffusion in amorphous [P_666,14_][DCA], [P_666,14_][SCN], and [P_666,14_][TCM] is thousands of times faster when compared to 1000s (log τ_σ_(*T*_*g*_) = 3) observed for all aprotic ILs examined so far.^30^ Specifically, log τ_σ_(*T*_*g*_) = −0.14^SCN^, −0.36^DCA^, and −0.38^TCM^ (for heating scans), and importantly, it is sensitive to the thermal history of the sample. That is, different values of log τ_σ_(*T*_*g*_) are obtained for slowly cooled and quenched IL. As shown in the zoomed image in Figure 5a, when liquid 2 of [P_666,14_][DCA] and [P_666,14_][SCN] is cooled slowly, it enters a glassy state at τ_σ_ much longer than it is for quenched material. Furthermore, the slow cooling of these ILs brings a glass of much higher apparent activation energy (Figure 5b). Thus, two different glassy states can be obtained within a single-component material. This phenomenon, called polyamorphism, gives a unique possibility to tune the properties of disordered electrolytes. However, it has never been observed before for ionic systems. From this point of view, it is interesting to note that in contrast to ILs containing [DCA] and [SCN] anions, the liquid–glass transition occurs at a single log τ_σ_(*T*_g_) for quenched and slowly cooled [P_666,14_][TCM]. This suggests that the self-assembly of alkyl chains in [P_666,14_]-ILs depends on the anion and brings differences in conductivity mechanisms between examined systems. However, fast charge transport, to some extent independent of structural rearrangements, is expected in supercooled liquid 2 and in the vicinity of the liquid-glass transition of [P_666,14_][DCA], [P_666,14_][SCN], and [P_666,14_][TCM]. To verify this hypothesis, there is a need for a direct comparison between the time scale of charge transport and structural dynamics, τ_α_, under the same conditions.

**Figure 6:**
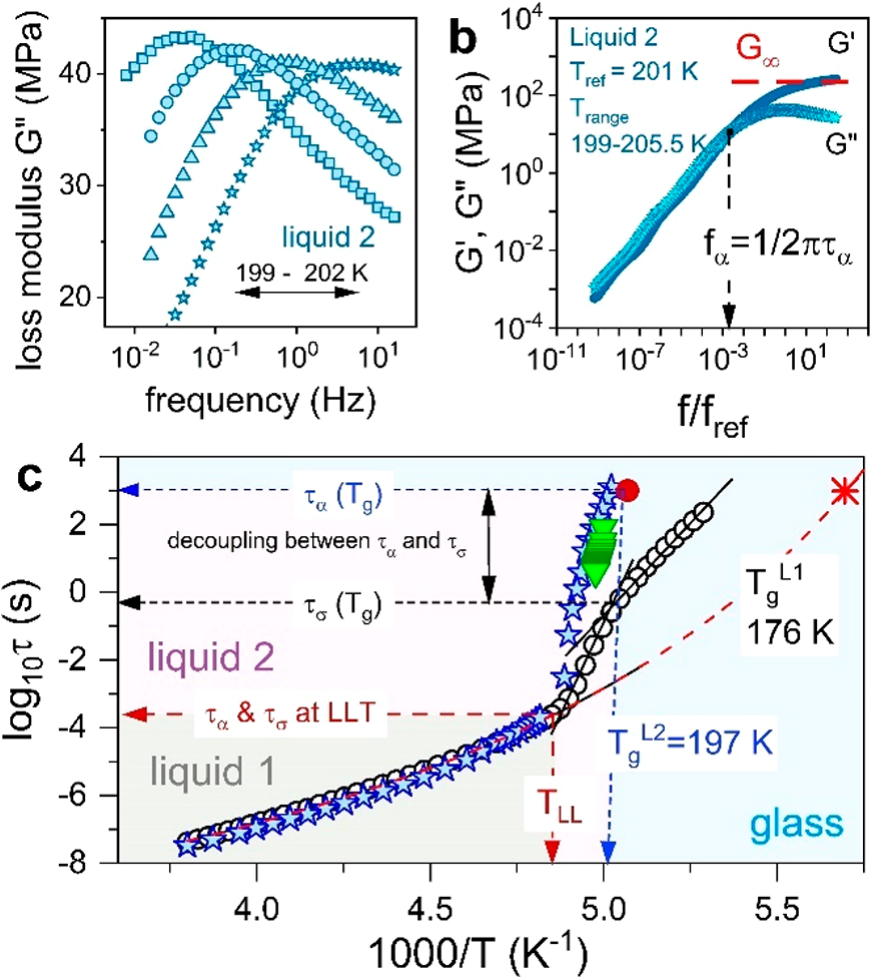
Panel a presents the mechanical response of [P_666,14_][DCA] recorded in supercooled liquid 2 and presented in the form of loss modulus peaks *G*″(*f*). In panel b, the mechanical data recorded at various temperatures in liquid 2 have been superimposed to each other and form the so-called masterplot. (c) Direct comparison between conductivity relaxation times (open circles) obtained on heating and structural relaxation times determined from rheology (blue stars) and TMDSC (green triangles) in supercooled liquids 1 and 2 and glass for [P_666,14_][DCA]. The red point denotes *T*_g_ from standard DSC. The red star presents the predicted value of *T*_g_ for the liquid 1 state.

Two experimental techniques were used to determine the time of structural motions: temperature-modulated DSC (TMDSC) and rheology. The experiments have been performed for [P_666,14_][DCA], and the representative results of mechanical measurements are illustrated in Figure 6a. In analogy to dielectric data, the frequency dependence of shear loss modulus *G*″ forms a well-resolved peak; the intersection of *G*′(*f*) and *G*″(*f*) gives the structural relaxation time τ_α_ (Figure 6b). Since the *G*″(*f*) peaks are well-identified only within four decades from the liquid–glass transition, the Maxwell relation τ_α_ = η/*G*_∞_ was employed to convert η(*T*^–1^) data to τ_α_(*T*^–1^) and thereby probe the structural dynamics of liquid 1. From Figure 6c, it becomes evident that the mechanical α-relaxation of [P_666,14_][DCA] follows the VFT law in liquid 1 and steeply increases at the temperature of LLT: over 8 K, there is a six-decade change in the value of τ_α_. Consequently, recalling condensed matter physics terminology, one can state that liquid 2 is much more fragile than liquid 1. Notably, an opposite conclusion was drawn by H. Tanaka for molecular liquid TPP,^31^ who explicitly identified LLT as fragile-to-strong transition.

An important conclusion is drawn from the direct comparison of the temperature dependencies of τ_α_ and τ_σ_, obtained for [P_666,14_][DCA]. In supercooled liquid 1, these two time scales are nearly identical, implying that the charge transport requires the diffusion of entire molecular units. Such a vehicle mechanism characterizes all aprotic ILs studied so far.^32^ Upon cooling below *T*_LLT_, the time scales of τ_α_ and τ_σ_ start to diverge: the structural motions become slower than the time scale of conductivity relaxation, and the most significant difference (decoupling) between these two variables, noted as *R*, is seen at *T*_g_, where *R* = log τ_α_(*T*_g_) – log τ_σ_(*T*_g_) = 3.1. Thus, when the structural relaxation time of the glassy phase is on the order of 1000 s, which gives [P_666,14_][DCA] mechanical properties of a solid, fast charge transport still occurs and takes around 1 s (log τ_σ_(*T*_g_) = 0). This decoupling occurs for all three [P_666,14_]^+^-based ILs and only slightly depends on the anion size. Such a phenomenon has never been reported for aprotic ILs or any other material revealing self-assembly or LLT, which raises an intriguing question about the mechanism behind this observation.

Taking into account the chemical structure of examined ILs and their self-assembly behavior, one can expect that in liquid 2, long alkyl chains of cations form a skeleton that contributes substantially to structural dynamics. At the same time, anions are free to move through created channels and thus are responsible for charge transport. Such a scenario has been previously described for polymerized ionic liquids, in which anions travel easily within channels of the covalently bonded, cationic polymer backbone.^33,34^ From this point of view, [P_666,14_][DCA], [P_666,14_][SCN], and [P_666,14_][TCM] in their liquid 2 state seem to act as single-ion conductors. To verify this hypothesis, we used [P_666,14_][DCA] as a reference system and determined the translational diffusion of cations in the liquid 2 state using ^1^H NMR relaxometry. We found that the *D*_trans_ of [P_666,14_]^+^ cations slows below *T*_LL_ and practically does not change with a further temperature decrease. *D*_trans_ at 204 K was found to be equal to 7.32 × 10^–14^ m^2^/s and fluctuates within the error of 2.82 × 10^–15^ m^2^/s in the liquid 2 state.

To provide more detailed insight into charge transport in the studied systems, we have performed high-pressure experiments.

### Compression through the Liquid–Liquid and Liquid–Glass Transitions

**Figure 7:**
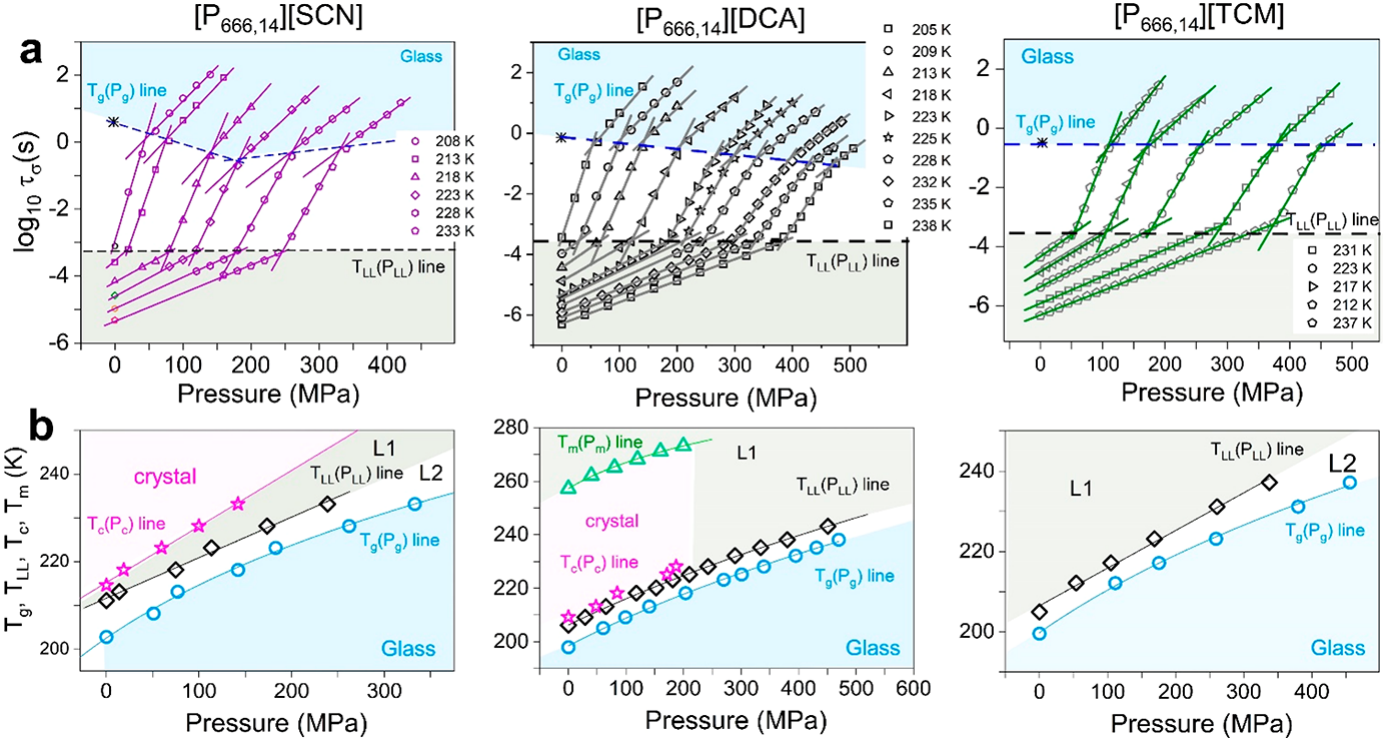
High-pressure data of [P_666,14_][SCN], [P_666,14_][DCA], and [P_666,14_][TCM] (from left to right). Panel a presents the pressure dependence of conductivity relaxation time measured at various *T*. Solid lines denote fit of the Arrhenius equation to experimental data. Dashed lines separate liquid 1 from liquid 2 (black dashed line) and liquid 2 from glass (blue dashed line). (b) *T*_LL_ and *T*_g_ as a function of *P* is presented. The color areas on panels a and b denote the liquid 1 phase (gray) and glass region (blue) and crystalline state (pink). L1 denotes liquid 1, while L2 denotes liquid 2.

The experimental protocol of high-pressure measurements has been described in the Materials and Methods section. The representative spectra recorded for [P_666,14_][DCA] in the pressure range 0.1–200 MPa are shown in Figure S4, while the isothermal conductivity relaxation times plotted as a function of pressure are presented in Figure 7a. In analogy to ambient pressure results, every *τ*_σ_–*P* dependence obtained for [P_666,14_][TCM], [P_666,14_][SCN], and [P_666,14_][DCA] reveals two kinks: first at *τ*_σ_ ≈ 3.5 ms separating two supercooled liquids and, second, being a manifestation of liquid-to-glass transition. Defining *P*_LL_ and *P*_g_ as the pressures at which the log* τ*_σ_ rapidly changes behavior, the *T*_LL_(*P*_LL_) and *T*_g_(*P*_g_) dependences were obtained for the three studied ILs (Figure 7b). In addition, the *T*–*P* conditions of cold crystallization have been included for [P_666,14_][SCN] and [P_666,14_][DCA]. Finally, the melting line *T*_m_(*P*_m_) for [P_666,14_][DCA] has been determined (Figure S5).

A closer inspection of Figure 7a reveals different pressure behaviors of ion dynamics in each examined IL. In [P_666,14_][TCM], both liquid–liquid and liquid–glass transitions occur at isochronal conditions: log* τ*_σ_*(T*_LL_*,P*_LL_*)* = −3.5 and log *τ*_σ_(*T*_g_*,P*_g_) = −0.5. In [P_666,14_][DCA], there is a continuous shift of *τ*_σ_(*P*_g_) toward shorter relaxation times with increasing pressure. Finally, in [P_666,14_][SCN], there is a clear minimum in the *τ*_σ_(*P*_g_) = *f*(*P*) dependence, around 170 MPa. Furthermore, for every *P*_g_ value, *τ*_σ_ is larger than 1000 s. Note that the time scale of structural relaxation is isochronal (τ_α_ ≈ 1000 s) regardless of *T*–*P* thermodynamic conditions.^35^ Thus, the examined phosphonium ILs are characterized by pressure-tunable fast charge transport, decoupled from structural relaxation, and governed by anion size. It can be speculated that for relatively small anions, [SCN]^−^ and [DCA]^−^, an increase in pressure results in better packing of cation alkyl chains and consequently provides more channels for anion motions. In this scenario, the diffusion of anions becomes faster at elevated pressures, which is visualized as a shorter log *τ*_σ_(*T*_g_*,P*_g_). However, above the pressure limit of 180 MPa, the [SCN]^−^ slows due to the reduced free volume, *V*_free_. The same effect is expected for [P_666,14_][DCA]; however, it probably occurs above the experimentally available pressure range. Further increase in anion size, in turn, makes the alkyl chain arrangements more difficult, which results in irregular channels for anion transport. In this case, anions still move faster than cations, making the system decoupled; however, squeezing does not affect it much, making log* τ*_σ_(*T*_g_*,P*_g_) pressure independent.

### MD Simulations of the Charge Transport Mechanism

**Figure 8:**
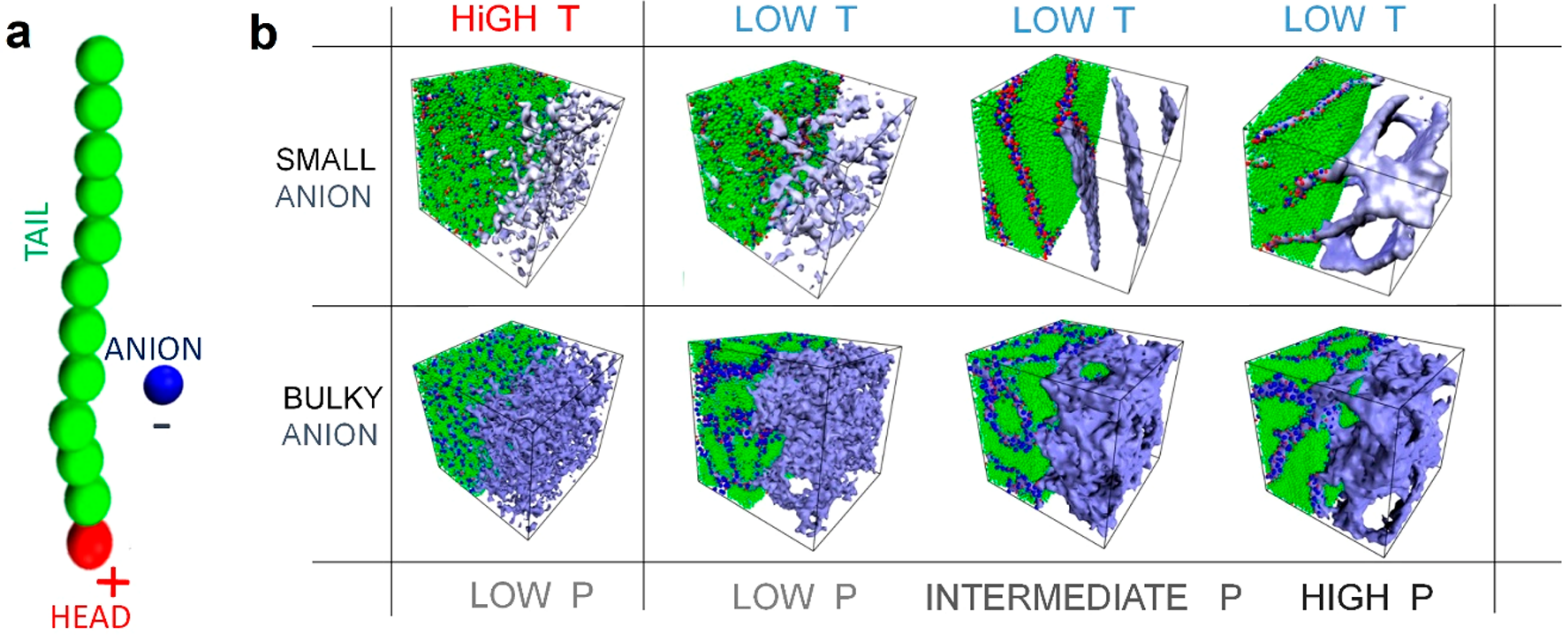
MD simulations snapshots. Panel a presents a single amphiphilic cation molecule with its counterion. Panel b illustrates IL morphologies under various *T*–*P* conditions. Columns present the molecular structure of the IL obtained at high (*T* = 5) and low (*T* = 2) reduced temperature and its variation with increased reduced pressure *P* (from low *P* = 2 through intermediate *P* = 5 to high *P* = 10). Rows display results for two different anion-to-cation size ratios, *R*_a_/*R*_c_ (small anion with *R*_a_/*R*_c_ = 1 and bulky anion with *R*_a_/*R*_c_ = 2). Isosurface representation of ionic channels density consisting of head groups and anions is displayed in blue.

MD simulations have been performed to verify the proposed mechanism of charge transport (see Supporting Information for details).^36−41^ To mimic the architecture of the phosphonium ILs, a simple bead–spring coarse-grained model was employed, consisting of an amphiphilic molecule containing a positively charged head connected to a stiff, neutral tail and a negatively charged free counterion (Figure 8a). Since recently we found that the 14-carbon chain is critical to observe LLT and that shortening the other three tails makes the LLT better detectable, we have been omitted the latter in MD simulations.^42^ The morphologies at high and low reduced temperatures and various pressures have been considered for two different anion-to-cation size ratios. At high temperatures, independent of the *R*_–_/*R*_+_ ratio, thermal fluctuations were dominant, yielding an isotropic structure of ILs. Upon a decrease in temperature, the cations tend to form nanostructures with different morphologies depending on applied pressure *P* and anion size. For small anions, an increase in pressure induces a morphological transition from weakly ordered aggregates composed of ionic pairs or triplets to lamellar-type phases.^16^ In contrast, for bulky anions, interconnected phases with continuous 3D curvature emerge regardless of *P*. Notably, the diffusivity of cations *D*_+_ is negligible in all examined cases (they are almost immobile, *D*_+_ ≈ 0), while anion dynamics is strictly related to the IL phase and applied pressure. Larger anions favor isotropic diffusivity, decreasing with pressure, whereas *D*_*–*_ varies nonmonotonically with pressure for smaller anions. Namely, the random distribution of small cation clusters leads to isotropic diffusivity of anions at low pressure, while lamellar-type phases, obtained at higher pressures, favor anisotropic diffusivity, making the anions transport 5 times faster along the lamellar plane. A pressure increase brings a further decrease in *D*_–_ (Figure S6).

## Conclusions

Here, we focused on the charge transport mechanism of three phosphonium ionic liquids comprising the same large amphiphilic cation with long, intertwined nonpolar alkyl chains and much smaller anions, charge-balanced by the cationic phosphonium centers. Our studies reveal that upon isothermal compression and isobaric cooling, the examined phosphonium ILs [P_666,14_][SCN], [P_666,14_][DCA], and [P_666,14_][TCM] transform from one liquid state to another, differing in self-assembly behavior, viscosity, and charge transport mechanism. The comparative analysis between the time scales of ion diffusion (τ_σ_) and structural dynamics (τ_α_) shows that charge transport is fully controlled by viscosity in liquid 1, that is, at *T* > *T*_LL_ and *P* < *P*_LL_. In contrast, liquid 2 has a nanostructure that facilitates charge transport decoupled from structural dynamics. Long alkyl chains of the cations are partially frozen in nonpolar domains while anions move swiftly through the created channels. From this point of view, the supercooled liquid 2 phase in [P_666,14_]^+^ ILs seems to mimic single-ion conductors such as polymerized ionic liquids. The self-assembled nanostructures of liquid 2, allowing fast ion transport, can be fine-tuned by sample thermal history, anion size, and compression. When quenching and slow cooling are applied, two different glasses differing in the time scale of ion motions can be obtained from the liquid 2 state. That is, in both disordered solids, the charge transport is independent of structural dynamics; however, in the one obtained by quenching, the decoupling is more pronounced compared to the slowly cooled system. This phenomenon, called polyamorphism, observed for the first time in ILs, gives a unique possibility to tune the properties of disordered electrolytes. Furthermore, a decrease in anion size brings nonmonotonic behavior of decoupling at elevated pressure. That is, τ_σ_(*P*_g_) reveals a minimum accompanied by diffusivity changes from isotropic to anisotropic character, and the latter facilitates anion transport along the lamellar-type plane. For bulky anions, interconnected phases with 3D continuous curvature emerge regardless of *P* and make τ_σ_(*P*_g_) constant. These results pave the way for a better understanding of self-organization in ILs and hence control the charge transport mechanism in ion-containing systems. The self-assembly-based charge transport mechanism discovered here offers a new approach for fine-tuning transport properties of ILs and other fluids with ordered nanostructures, which could profoundly impact emerging technologies associated with ionic liquids as conductive soft materials.

## Supporting Information Available

Details on MD simulations and supplementary dielectric and calorimetric results (PDF)

## Abbreviations

LLT: liquid–liquid transition
