# Supplementary material for: Self-Assembled Nanostructures in Aprotic Ionic Liquids Facilitate Charge Transport at Elevated Pressure
Source: ACS Appl Mater Interfaces. 2023 Aug 9;15(33):39417–25. doi: 10.1021/acsami.3c08606 (PMC10450691; doi:10.1021/acsami.3c08606)
Supplement: Supplementary file 1 — am3c08606_si_001.pdf [file am3c08606_si_001.pdf]

Supporting Information

Self-assembled nanostructures in aprotic ionic liquids facilitate charge transport at elevated pressure

Beibei Yao[1], Marian Paluch[1], Jaroslaw Paturej[1], Shannon McLaughlin,[2] Anne McGrogan,[2] Malgorzata Swadzba-Kwasny[2], Jie Shen[3,4], Beatrice Ruta[3,4], Martin Rosenthal[5,6], Jiliang Liu[4], Danuta Kruk[7], Zaneta Wojnarowska[1]*

[1]Faculty of Science and Technology, Institute of Physics, University of Silesia in Katowice, 75 Pułku Piechoty 1A, 41–500 Chorzów, Poland

[2]The QUILL Research Centre, School of Chemistry and Chemical Engineering, The Queen's University of Belfast, David Keir Building, Stranmillis Rd, BT9 5AG Belfast, NI, UK.

[3]Institut Neel, 38000 Grenoble, France. 2

[4]ESRF- The European Synchrotron, CS 40220, 38043 Grenoble, France

[5]Department of Chemistry, KU Leuven, Celestijnenlaan 200F, Box 2404, B-3001 Leuven, Belgium

[6]Dual Belgian Beamline (DUBBLE), European Synchrotron Radiation Facility, 71 avenue des Martyrs, CS 40220, 38043 Grenoble Cedex 9 France

[7]Faculty of Mathematics and Computer Science, University of Warmia and Mazury in Olsztyn, Sloneczna 54, Olsztyn PL-10710, Poland

This PDF file includes:

Supplementary Text
Figs. S1 to S6
Tables S1
References (1 to 7)

Supplementary Text

Supplementary Methods

Trihexyl(tetradecyl)phosphonium chloride, [P_{66614}]Cl, was kindly provided by Solvay. Sodium tricyanomethanide was bought from TCI Chemicals. All other chemicals were purchased from Sigma-Aldrich and used as received. XRF analysis was performed on a Rigaku NEX QC+ QuantEZ High-Resolution Energy Dispersive X-ray Fluorescence (EDXRF) Spectrometer. NMR spectra were recorded on either a Bruker Avance III 400 MHz spectrometer or a Bruker Avance II DPX 600 MHz spectrometer. [$P_{666,14}$][DCA] was supplied from Ionitec.

[$P_{666,14}$][SCN]. Trihexyl(tetradcyl)phosphonium chloride [$P_{666,14}$]Cl (0.010 mol eq.) and K[SCN] (0.013 mol eq.) were separately added to 25 cm^3 deionised water (18.2 MΩ.cm) deionised water (total 50 cm^3) and then combined in a round-bottomed flask (250 cm^3), resulting in the formation of a biphasic liquid system; the mixture was left to react (1 h, room temperature, 600 rpm). The aqueous layer was separated, and the organic layer was collected and washed, firstly with deionised water (10 cm^3) and then dichloromethane, DCM (10 cm^3). Subsequent washes were performed with solution of K[SCN] in deionised water (18.2 MΩ.cm). Final three washes were performed with deionised water (18.2 MΩ.cm) until no chloride could be detected with XRF. Subsequently, DCM was removed *via* rotary evaporation (30 min, 35 °C) and the ionic liquid was dried under high vacuum (12h, 70 °C, 10^{-2} mbar). XRF analysis of [$P_{666,14}$][SCN] confirmed chloride content was below the detectable limit. ^{1}H, ^{13}C and ^{31}P NMR spectra of the ionic liquid were recorded in d_6-DMSO.

[$P_{666,14}$][TCM]. Trihexyl(tetradecyl)phosphonium chloride [$P_{666,14}$]Cl (0.010 mol eq.) and sodium tricyanomethanide, Na[TCM] (0.013 mol eq.) were separately added to 25 cm^3 deionised water (18.2 MΩ.cm) (total 50 cm^3) and then combined in a round-bottomed flask (250 cm^3), resulting in the formation of a biphasic liquid system; the mixture was left to react (1 h, room temperature, 600 rpm). The aqueous layer was separated, and the organic layer was collected and washed, firstly with deionised water (18.2 MΩ.cm) (10 cm^3) and then dichloromethane, DCM (10 cm^3). Six subsequent washes were performed with solution of Na[TCM] in deionised water (18.2 MΩ.cm). Final three washes were performed with deionised water (18.2 MΩ.cm) until no chloride could be detected with silver nitrate solution. Subsequently, DCM was removed *via* rotary evaporation (30 min, 35 °C) and the ionic liquid was dried under high vacuum (12h, 70 °C, 10^{-2} mbar). XRF analysis of [$P_{666,14}$][TCM] recorded a chloride content of 89.3 ppm with a lower detection limit (LLD) of 2.80 ppm. ^{1}H, ^{13}C and ^{31}P NMR spectra of the ionic liquid were recorded in d_6-DMSO.

Prior to the measurements, the samples were dried under vacuum at 90 °C for 24 h. The water content detected using the Karl Fischer method, was around 500 and 300 ppm before and after drying, respectively.

Coarse-grained model, simulation methodology and numerical results.

Molecular dynamics simulations of ionic liquids comprised of amphiphile molecules were performed using a bead-spring model at coarse-grained resolution. We adopted the numerical model that was used in the previous study of conductivity in dry ionic liquids [1]. An individual amphiphile molecule is composed of a positively charged head (cation) linked to a stiff tail of $N_t = 11$ adjacently connected neutral beads. The system electroneutrality was maintained by adding negatively-charged counterion beads to compensate for each charged cation on the amphiphile. The non-bonded interactions between beads i and j, separated by a distance r , are accounted by the truncated and shifted expanded Lennard-Jones (LJ) potential [2]

$$V^{LJ}(r) = \begin{cases} 4\epsilon\left[\left(\frac{\sigma}{r-\Delta_{ij}}\right)^{12} - \left(\frac{\sigma}{r-\Delta_{ij}}\right)^{6}\right] + \delta_{ij} & r \le r_{ij}^{\text{cut}} + \Delta_{ij}, \\ 0 & r > r_{ij}^{\text{cut}} + \Delta_{ij}. \end{cases} \quad (1)$$

In the above equation $\epsilon = k_B T$ is the parameter which controls strength of the LJ potential, where k_B is the Boltzmann constant and T is the absolute temperature. The potential of (Eq. 1) is a standard 12-6 LJ potential shifted to the right by the parameter Δ_{ij} which allows to account for interactions between beads of different size. In (Eq. 1) the potential shift for tail-tail Δ_{tt}, tail-cation Δ_{tc} and cation-cation Δ_{cc} pairs is $\Delta_{tt} = \Delta_{tc} = \Delta_{cc} = 0$. This yields the equal size of tail R_t and cation R_c beads which is set to: $R_t = R_c = \sigma$. The potential shift for tail-anion Δ_{ta}, cation-anion Δ_{ca} and anion-anion Δ_{aa} pairs is: $\Delta_{ta} = \Delta_{ca} = (R_a - \sigma)/2$ and $\Delta_{aa} = R_a - \sigma$, where R_a is the size of anion. In this study we consider two diameters of anions, i.e. $R_a = \sigma$ (denoted as small anions) and 2σ (denoted as bulky anions). The cutoff distance r_{ij}^{cut} in (Eq. 1) is adjusted to model hydrophobic and hydrophilic interactions between beads. To account for hydrophobicity between tail beads we take $r_{tt}^{\text{cut}} = 6\sigma$. The same cutoff was used for the interactions between cation heads $r_{cc}^{\text{cut}} = 6\sigma$. All other pairs of beads interact through the LJ potential with a cutoff $r_{tc}^{\text{cut}} = r_{ta}^{\text{cut}} = r_{ca}^{\text{cut}} = r_{aa}^{\text{cut}} = 2^{1/6}\sigma$. This choice provides a repulsive LJ force between hydrophobic tails and hydrophilic head groups and anions. The quantity δ_{ij} in (Eq. 1) is chosen such that $V^{eLJ}\left(r_{ij}^{\text{cut}} + \Delta_{ij}\right) = 0$.

The connectivity between two adjacent chain monomers is maintained by the finite extension nonlinear elastic (FENE) potential [3,4]

$$V^{FENE} = -\frac{1}{2} k_F r_F^2 \ln\left[1 - \left((r - \Delta_{ij})/r_F\right)^2\right]. \quad (2)$$

where $k_F = 30\epsilon/\sigma^2$ is the bond spring-constant and $r_F = 1.5\sigma$ is the maximum bond length. Depending on the bead size the spring extends up to $r_F + \Delta_{ij}$.

In our experiments tails are expected to be stiff. To account for large persistence length of tails, in simulations we introduced bond-bending potential which acts on three consecutive tail beads. The bending potential reads

$$V^B = k_\theta(\theta - \theta_0)^2, \quad (3)$$

where $k_\theta = 10\epsilon/\text{rad}^2$ is the bending stiffness which yields persistence length on the order of the tail size. In (Eq. 3) θ is the angle between two subsequent tail bond vectors and $\theta_0 = 180^o$ is the reference angle.

The Coulombic interactions were introduced by assigning to each cation head a rescaled charge of $q = +e/\sqrt{4\pi\epsilon_0\sigma\epsilon}$ where e stands for the elementary charge and ϵ_0 is the electric permittivity of the vacuum. Each anion carries an opposite charge of $-q$. The short-range electrostatic interactions between any two charged particles are calculated via

$$V^C(r) = \pm\epsilon\frac{l_B}{r} \qquad r < r_e \quad , \tag{4}$$

where the Bjerrum length $l_B \equiv q^2\sigma/\epsilon_p$ determines the strength of electrostatic interactions and ϵ_p is the relative dielectric constant of the medium. In our simulations we set $l_B = 10\sigma$ corresponding to $\epsilon_p = 80$ which is in accordance with dielectric constant reported for dense polymeric melts [1,5]. The electrostatic cut-off distance r_e in Eq. (4) was set to $r_e = 10\sigma$. Above r_e longer-range electrostatic interactions are calculated via Particle-Particle-Particle Mesh (PPPM) Ewald method and the error tolerance for force was set to $10^{-4}\epsilon/\sigma$.

The LAMMPS package [6] was utilized to perform molecular dynamics simulations in NPT ensemble using Nosé-Hoover barostat. Simulations were carried out in a cubic box with periodic boundary conditions. Simulations were run with a timestep of $\delta t = 0.005\tau$, where $\tau = \sigma\sqrt{m/\epsilon}$ is the time unit. Bead masses were set to $m = 1$ for all tail monomers including cation head. The mass of anion was set proportional to its diameter, i.e. through relationship: $m_a = m(R_a/\sigma)^3$.

Each system was comprised of 5400 amphiphile molecules and equal amount of counterions. For each individual system we firstly carried out simulations at high temperature $T = 5$ to obtain isotropic phase. The morphologies of ionic liquids at low temperature were produced using step-annealing procedure in which we slowly cooled down the system from high $(T = 5)$ to low $(T = 2)$ temperature at prescribed pressure P. The temperature step size during annealing was $\Delta T = 0.25$. We consider three values of P in this study denote as low ($P = 2.5$), intermediate ($P = 5.0$) and high ($P = 10\ \sigma^3/\epsilon$) pressure, respectively. Each system was simulated at least $5 \cdot 10^7$ time steps to ensure thermodynamic stability of the observed phase. We also tested different starting configurations of ionic melts at high temperature to exclude possible effects of the initial configurations on the final assembled morphologies. We found no influence of starting configuration on the resulting ionic liquid assemblies.

In Fig. S6 we display MD data for diffusivity D_a of small anions with anion-to-cation size ratio $R_a/R_c = 1$ (depicted as squares) and bulky anions with $R_a/R_c = 2$ (displayed as circles) measured for ionic liquid morphologies obtained at different pressure conditions. The values of D_a were calculated from the mean-squared displacement of anions. The relative size of anions strongly influences pressure-dependent diffusivity profiles. For anions of the same size as cations (R_a/R_c=1) nonmonotonic behavior is observed. Here, the increase in D_a between pressure $P = 2.5$ and $5\ \sigma^3/\epsilon$ is due to morphological transition of ionic liquid from weakly ordered aggregates composed of ionic pairs or triplets to ordered lamellar phases. The lamellar phases obtained for $P > 5\sigma^3/\epsilon$ have highly anisotropic diffusivity which is signaled by an increase in the ratio $D_{||}/D_{\perp}$ of parallel to perpendicular components of D_a (cf. inset of Fig. S6). The decrease in D_a visible

between $P = 5$ and $10\ \sigma^3/\epsilon$ is caused by an increase in the overall bead density in the whole system ($\approx 0.9\sigma^{-3}$) which significantly hinders anion dynamics. Larger size polarity between anion and cation (i.e. for $R_a/R_c = 2$) induces 3D inter-connected morphological phases of ionic liquids and leads to monotonic decrease of D_a with increasing P. Here diffusivity is isotropic ($D_{||}/D_{\perp} \approx 1$) irrespectively of applied P (see inset of Fig. S6).

Table S1

Thermodynamic properties of studied ILs.

	**Anion**		
	**$[SCN]^-$**	**$[DCA]^-$**	**$[TCM]^-$**
T^{c}_{LLT} (K)	213.3	209.04*; 209.1	207.4
$-\Delta H^{c}_{LLT}$ (Jg^{-1})	11.4	10.3*; 9.2	8.1
T^{h}_{LLT} (K)	207.1	204*; 203.8	204.8
ΔH^{h}_{LLT} (Jg^{-1})	11.6	11.5*; 9.9	8.6
T_g (K)	197.7[a]; 199.1[b]	197.3[a]	197.7[a]; 196.3[b]
T_{LL}^{BDS} (K)	210.3	203	203.6
T_c (K)	254.3	231.7*, 240.6	-
$-\Delta H_c$ (Jg^{-1})	1.9	23.3*, 1.3	-
T_m (K)	272.3	254.2*, 256.2	-
ΔH_m (Jg^{-1})	5.0	24.1*, 1.3	-
ν (nm^3)	0.048	0.064	0.093
dT_g/dP ($KGPa^{-1}$)	144	112	118
dT_{LL}/dP ($KGPa^{-1}$)	109	104	93
dT_m/dP ($KGPa^{-1}$)	-	130	-

a: The glass transition temperature was determined from DSC measurements after 6h aging at 183.15K with the heating rate 10 K/min;
b: The glass transition temperature determined from dielectric measurements
*5 K/min; Others: 10 K/min
The van der Waals volume was calculated by using the Bondi method.

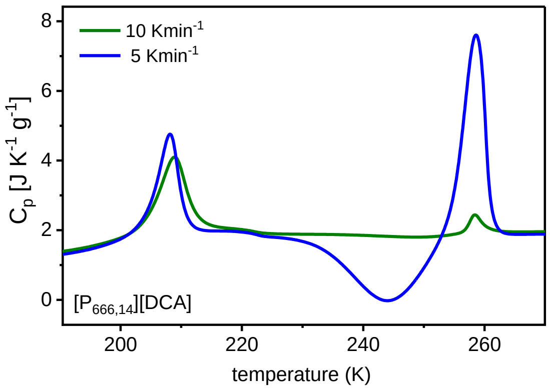

Figure S1

The DSC thermograms of $[P_{666,14}][DCA]$ obtained on heating with the rates of 5 and 10 $Kmin^{-1}$.

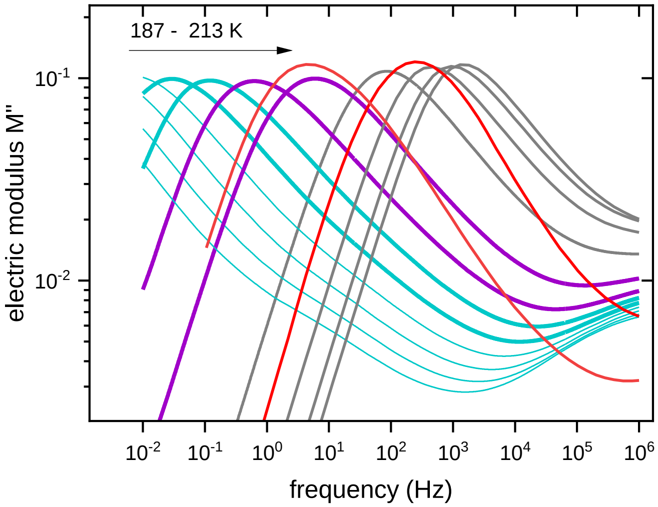

Figure S2

The dielectric data of $[P_{666,14}][DCA]$ obtained on heating from 187 to 213 K with the step of 2 K. Cyan lines are obtained in the glassy state, violet in liquid 2, grey in liquid 1 and the red lines denote crystalline state of examined sample.

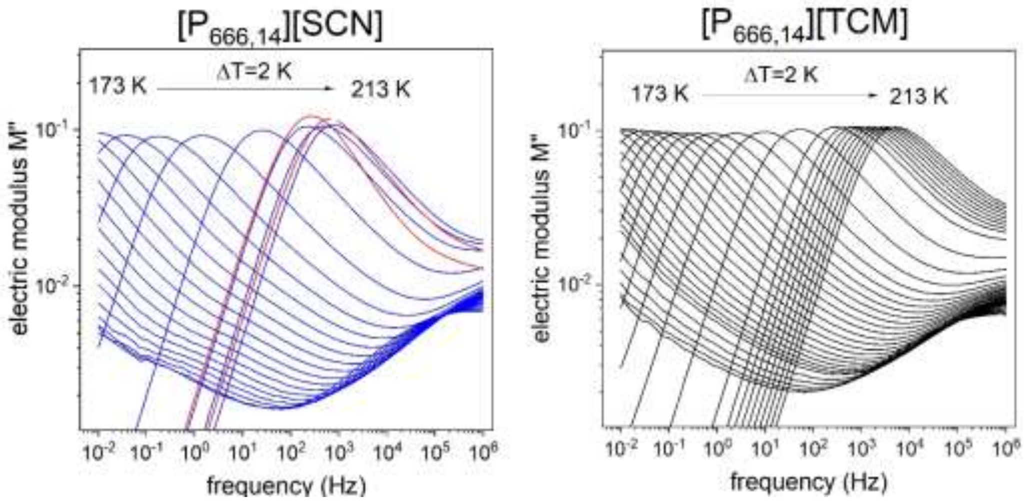

Figure S3

The dielectric data of $[P_{666,14}][SCN]$ and $[P_{666,14}][TCM]$ obtained on heating from 173 to 213 K with the step of 2 K. Red lines denote crystalline state of examined sample.

To quantitatively compare dielectric data of $[P_{666,14}][DCA]$, $[P_{666,14}][SCN]$ and $[P_{666,14}][TCM]$, the same experimental protocol, i.e., measurement on heating after preceding quenching, has been employed (see Fig. S3).

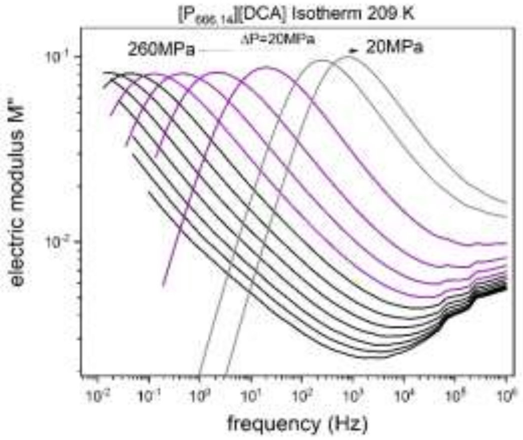

Figure S4

The dielectric spectra of $[P_{666,14}][DCA]$ obtained during decompression at 209 K. Black lines denote glassy state, violet-liquid 2 state, grey-liquid 1 state.

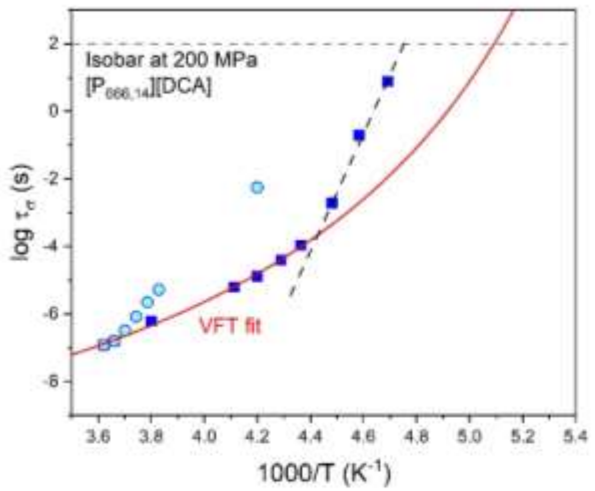

Figure S5
The conductivity relaxation times of [$P_{666,14}$][DCA] at 200 MPa (blue squares). The data in liquid 1 are parametrized by the VFT equation. Blue circles are obtained during the heating after crystallization at 228 K and 200 MPa. The melting point at 200 MPa was determined as the temperature where experimental data meet the VFT line.

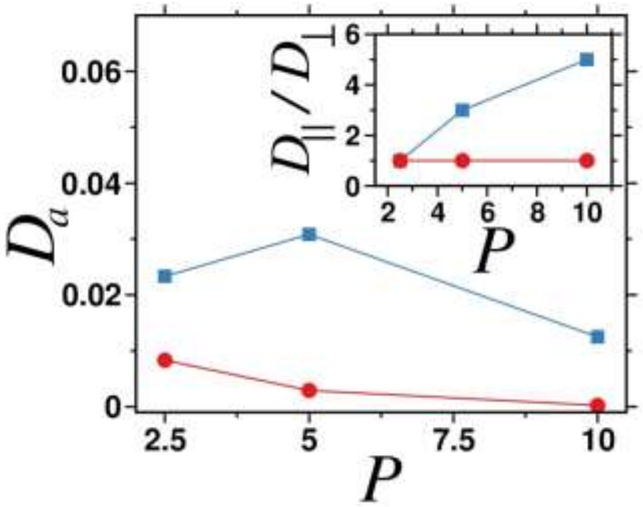

Figure S6
MD simulation data for diffusivity of anions D_a in ionic liquids at temperature $T = 2$ plotted as a function of imposed pressure P. Data displayed for different anion-to-cation size ratios $R_a/R_c = 1$ (squares) and $R_a/R_c = 2$ (circles). The inset shows the ratios $D_{||}/D_{\perp}$ of parallel to perpendicular components of diffusivity vs. P.

1. Erbas, A.; Olvera de la Cruz M. Morphology-enhanced conductivity in dry ionic liquids. *Phys. Chem. Chem. Phys.* **2016**, *18*, 6441-6450.

2. Smith, J. S.; Bedrov, D., Smith, G. D. A Molecular Dynamics Simulation Study of Nanoparticle Interactions in a Model Polymer-nanoparticle Composite. *Comput. Sci. Technol.* **2003**, *63*, 1599-2003.

3. Kremer, K.; Grest, G. S. Dynamics of Entangled Linear Polymer Melts: A Molecular-Dynamics Simulation. *J. Chem. Phys.* **1990**, *92* (8), 5057-5086.

4. Grest, G. S.; Kremer, K. Molecular Dynamics Simulation for Polymers in the Presence of a Heat Bath. *Phys. Rev. A* **1986**, *33* (5), 3628-3631.

5. D.R. Lide, *CRC Handbook of Chemistry and Physics*, 2004.

6. Plimpton, S. J. Fast Parallel Algorithms for Short-range Molecular Dynamics. *J. Comp. Phys.* **1995**, *117* (1), 1-19. (http://lammps.sandia.gov).

7. Humphrey, W.; Dalke, A.; Schulten, K. VMD: Visual Molecular Dynamics, *J. Molec. Graph. Model.* **1996**, *14* (1), 3
